# Systematic review on outcomes used in clinical research on autosomal recessive polycystic kidney disease—are patient-centered outcomes our blind spot?

**DOI:** 10.1007/s00467-021-05192-8

**Published:** 2021-08-12

**Authors:** Charlotte Gimpel, Max Christoph Liebau, Franz Schaefer

**Affiliations:** 1 Department of Medicine IV, Medical Center–University of Freiburg, Faculty of Medicine, University of Freiburg, Hugstetter Str. 55, 79106 Freiburg, Germany; 2 Praxis für Kinderkardiologie und Kindernephrologie, Medizinisches Versorgungszentrum des Klinikum Konstanz, Konstanz, Germany; 3 Department of Pediatrics, Faculty of Medicine and University Hospital Cologne, University of Cologne, Cologne, Germany; 4 Center for Molecular Medicine Cologne, Faculty of Medicine and University Hospital Cologne, University of Cologne, Cologne, Germany; 5 Division of Pediatric Nephrology, Center for Pediatrics and Adolescent Medicine, University Hospital Heidelberg, Heidelberg, Germany

**Keywords:** ARPKD, SONG initiative, Outcomes, Patient centered, Patient-reported outcome measures, Systematic review

## Abstract

**Background:**

Autosomal recessive polycystic kidney disease (ARPKD) is a rare severe hepatorenal disease. Survivors of pulmonary hypoplasia and patients with milder presentations often achieve long-term survival but frequently require kidney and/or liver transplantation.

**Objective:**

To examine the use of clinical, surrogate and patient-centered outcomes in studies on ARPKD with special attention to core outcomes of the Standardized Outcomes in NephroloGy project for children with chronic kidney disease (SONG-Kids).

**Data sources and study eligibility criteria:**

A systematic MEDLINE literature search identified 367 ARPKD studies published since 1990; however, of these 134 were excluded because they did not report any clinical outcomes (e.g. only histopathological, genetic, protein structure or radiological markers), 19 studies because they only included prenatal patients and 138 because they were case reports with ≤ 3 patients.

**Study appraisal:**

Seventy-six eligible studies were examined for study type, size, intervention, and reported outcomes by organ system and type, including all SONG-kids tier 1–3 outcomes.

**Participants:**

There were 3231 patient-reports of children and adults with ARPKD.

**Results:**

The overwhelming majority of studies reported clinical and surrogate outcomes (75/76 (98%) and 73/76 (96%)), but only 11/76 (14%) examined patient-centered outcomes and only 2/76 (3%) used validated instruments to capture them. Of the SONG-Kids core outcomes, kidney function was reported almost universally (70/76 (92%), infection and survival in three quarters (57/76 (75%), 55/76 (72%)) and measures of life participation (including neurological impairment) only rarely and inconsistently (16/76 (21%)).

**Limitations:**

Thirty studies (39%) were of low quality as they were either narrative case reports (n = 14, 18%) and/or patients with ARPKD were an indistinguishable subgroup (n = 18, 24%). Only 28 trials compared interventions, but none were randomized.

**Conclusions and implications:**

Studies that reported clinical outcomes in ARPKD usually covered the core outcome domains of kidney function, infections, and survival, but measures of life participation and patient-centered outcomes are distinctly lacking and require more attention in future trials.

**Graphical abstract:**

**Figure:**
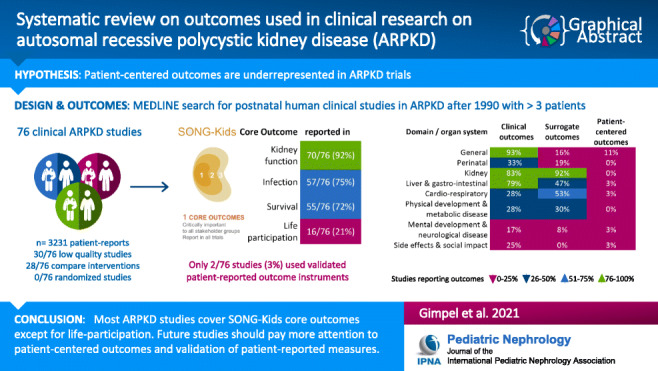
A higher resolution version of the Graphical abstract is available as Supplementary information

**Supplementary Information:**

The online version contains supplementary material available at 10.1007/s00467-021-05192-8.

## Background

Autosomal recessive polycystic kidney disease (ARPKD) is a rare severe hepatorenal disease. Antenatal disease can lead to intrauterine death or termination of pregnancy [1]. Kidney failure in the first year of life occurs in about 10% of live-born children, especially those with oligohydramnios and pulmonary hypoplasia [2], and the overall risk of childhood-onset kidney failure is about 50% [3]. The manifestations of portal hypertension due to hepatic involvement take more time to develop but become clinically relevant in up to 50% of children [3]. ARPKD has become one of the most common indications for childhood combined liver–kidney transplantation [4].

The low incidence of ARPKD (of about 1 in 20,000 [5]) has made systematic research difficult, and there are no randomized controlled trials to date. With an increasing awareness of individuals with hepatic-dominant phenotype [6] as well as improvements in neonatal survival [7, 8], questions of long-term management are becoming increasingly relevant. On the other hand, clinical and basic science research in the field of autosomal dominant polycystic kidney disease (ADPKD) has been very dynamic in recent years and the overlapping pathophysiological elements may expedite the development of therapeutic agents for ARPKD [9], with two trials registered very recently [10, 11]. To define therapeutic targets in ARPKD, it is necessary to validate end-points and clinical outcomes and establish accurate ways to measure them and to achieve consensus on core outcomes. The need to define suitable outcome measures applies both to clinical and surrogate measures as well as to patient-reported outcome measures (PROM). The International Rare Diseases Research Consortium (IRDiRC) stresses that rare diseases are not exempt from the need to develop and use PROMs and that these efforts should be started early on [12]. Incorporation of patients’ perspectives into clinical trials is very important to ensure long-term personal and societal benefit of new treatments [13]. Even though most pediatric nephrologists can testify to the major impact of ARPKD on their patients’ health-related quality of life and the significant burden for their families, there is very little quantitative or qualitative research on patients’ experience and attitudes.

As a starting point, the Standardized Outcomes in Nephrology (SONG) initiative has recently provided valuable insights into the perspectives of patients with kidney disease. The SONG-Kids project focuses on children with all-cause chronic kidney disease [14] and has provided a systematic literature review [15], international patient focus groups [16], Delphi surveys among patients and health professionals [17], and a consensus workshop [18] on core outcomes for this patient group. Additionally, the SONG-PKD project has provided a set of core outcomes for adults with ADPKD with thorough involvement of all stakeholders [19]. The SONG projects rank outcome domains into three tiers: core outcomes (critically important to all stakeholders), middle-tier outcomes (critically important to some stakeholders), and outer-tier outcomes (important to some stakeholders). Many of the outcomes in all three tiers can be considered patient centered, such as life participation and the ability to travel or work; but validated pediatric patient-(or proxy-)reported outcome measures are only available for some, such as scales for pain, anxiety, or health-related quality of life.

The aim of this study was to assess which clinical outcomes, surrogate measures, and patient-reported outcomes have been used in ARPKD research to date, with special attention to outcomes deemed relevant by the SONG-kids and SONG-PKD projects and validated patient-centered outcome instruments. This is intended to uncover our “blind spots” which need development before ARPKD research can enter the next phase of improving long-term patient outcomes.

## Methods

### Search strategy and study assessment

**Fig. 1:**
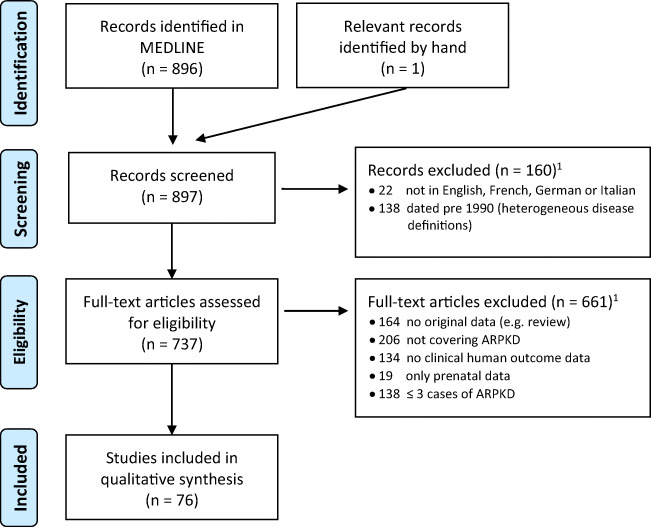
Selection of eligible papers and reasons for exclusion. ^1^Only one reason for exclusion per paper (sequential exclusion), for overall numbers see text. ARPKD: autosomal recessive polycystic kidney disease

Relevant publications were searched for in MEDLINE using the search term ((ARPKD) OR "autosomal recessive polycystic kidney disease" OR "congenital hepatic fibrosis" OR "hepatorenal cystic syndrome") AND (child* OR young adult OR adolescent* OR pediatric). The last search update was performed on 09 April 2021 yielding 897 studies. A flow diagram listing the number of excluded papers by reason is given in Fig. 1. Studies published prior to 1990 were excluded because there was significant heterogeneity regarding the definition of ARPKD (then mainly called infant polycystic kidney disease). Foreign language papers after 1990 were mainly in Chinese. Full-text manuscripts were assessed for exclusion criteria sequentially; thus, *n* numbers in Fig. 1 refer to any further studies that were excluded. Overall, 192 publications did not contain new data because they were reviews, letters, trial descriptions, or position statements, and 292 did not cover ARPKD, either because they examined other cystic kidney diseases or congenital hepatic fibrosis without stating whether kidney involvement was present. Neonatal PKD with hyperinsulinism and other genetic phenocopies were also excluded, despite the similar phenotype, because of the distinct genotype. Studies which did not include human clinical outcome data (n = 143) mainly dealt with animal models (n = 70), histopathology and/or cell cultures (n = 31), genetic sequence analysis (n = 23), or protein structure (n = 3), but also 16 radiological studies in humans which did not present imaging findings in relation to outcomes. Because of the focus on patient-reported outcomes, studies only assessing prenatal features or outcomes were also excluded (27 studies overall). Over 40% of all ARPKD-papers with original data (151/367, 41%) were case reports with ≤ 3 postnatal ARPKD patients.

All eligible papers (n = 76) were searched for reported clinical, surrogate, and patient-centered outcome measures with special attention to those deemed important in the SONG-Kids project but extended to pre-/neonatal, hepatic, and pulmonary domains that reflect the multisystem nature of ARPKD.

### Statistical analysis

Data were stored and analyzed using Microsoft Excel. Group characteristics are given as mean and standard deviation for continuous variables. Group comparisons were performed using the Fisher exact test because many outcomes were reported infrequently. For both tests, a *p* value of < 0.05 was considered significant.

## Results

### Study characteristics

About two-thirds of the published original studies on ARPKD since 1990 (233/367 (63%)) assessed human outcome data, but two-thirds of these (157/233 (67%)) included only 3 or less patients after birth, leaving 76 studies for detailed analysis reported here.

There was a continual increase of research activity with 9 papers from 1990–1999 (0.9 per year), 17 from 2000–2009 (1.7 per year), 37 from 2010–2019 (3.7 per year), and 13 in 2020.

Of the 76 studies, 51 (67%) included only children, 2 (3%) only adults, and 23 (30%) both children and adults. Altogether there were 3231 patient reports, with a median sample size of 16 ARPKD patients (range 4–484; mean 43.1 ± 80 patients); however, some populations were reported on more than once and it was not possible to determine the underlying number of individual patients. Despite the exclusion of small case series, most studies still included fewer than 20 patients (n = 44, 58%) and only 7 studies (9%) more than 100 individuals with ARPKD.

Thirty-nine studies (51%) reported only on patients with ARPKD (with or without healthy volunteers). Of the remaining 37 papers which also studied other patients, results of the ARPKD-subgroup could be well identified in 11 (30%), were only partly identifiable in 8 (22%), and were not presented separately in 18 (49%). In 23 of 73 studies (30%), some or all patients had genetically confirmed disease. Despite the prior exclusion of studies with less than 4 ARPKD patients, 15 studies (20%) were still narrative case reports listing only relevant positive outcomes and 7 were partly narrative and partly systematic, leaving 54 (71%) studies with systematic reporting. This left only 46 (61%) “higher quality” studies which reported results (of the ARPKD subgroup) systematically (of which 32 (70%) in children only, 1 (2%) in adults only and 13 (28%) in adults and children).

Only 10 trials (13%) collected data prospectively, while 48 (63%) reported retrospective and 18 (24%) cross-sectional analyses. None of the 76 studies were randomized controlled trials, and only 28 compared different interventions, which included either different surgical treatments (n = 5: nephrectomy, portosystemic shunts, or percutaneous endoscopic gastrostomy), different modes of kidney replacement therapy (n = 5), different types of transplant surgery (n = 17), or different pharmacological treatments (n = 1: growth hormone). Only 17 of the 28 interventional trials (61%) used control groups, but 12 of the 48 non-interventional studies (25%) also had control groups.

There was a slight preponderance of studies with an emphasis on kidney manifestations of ARPKD (28/76, 38%), compared to gastroenterological manifestations (22/76, 29%), while 26 (34%) paid equal attention to both. Twenty-five studies (33%) included only patients without transplants, while 19 (25%) focused on patients after transplantation and 32 (42%) included both. About the same number of studies included some or only patients after kidney transplantation (33 (43%) and 13 (17%)), or after liver transplantation (32 (42%) and 5 (7%)).

### Outcomes by type and domain

The overwhelming majority of studies reported one or more clinical outcomes (75/76, 98%), or one or more surrogate outcomes (73/76, 96%), but only 11/76 (14%) reported a patient-centered outcome.

Table 1 lists an overview of the proportions of studies reporting clinical, surrogate, and patient-centered outcomes by organ-system or domain. While the renal and gastrointestinal domains were covered in most studies (kidney 71/76 (93%) and gastrointestinal 62/76 (82%)), outcomes from the domains of cardio-respiratory disease (47/76 (62%)), physical development, bone or metabolic disease (25/76 (33%), and mental development, neurological disease, or psychological impact (15/76 (20%)) were less frequent.

**Table 1:**
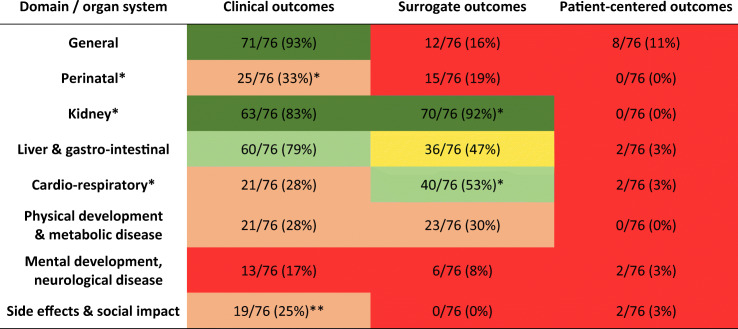
Number of ARPKD studies since 1990 reporting at least one outcome in different domains of clinical, surrogate, and patient-centered outcomes. Color-coding: red 0–20%, rose 21–40%, yellow 41–60%, light green 61–80%, green 81–100%. * and ** indicate that high-quality studies scored significantly better or worse than lower-quality studies in this area (see text for detailed values)

The higher-quality studies^1^ were significantly better than lower-quality studies at covering outcomes in three domains: all perinatal outcomes (23/46 (50%) vs. 5/30 (17%), *p* = 0.002), all kidney outcomes (46/46 (100%) vs. 25/30 (83%), *p* = 0.008), and all cardio-respiratory outcomes (34/46 (74%) vs. 13/30 (43%), *p* = 0.005). This was mainly due to better coverage in clinical perinatal outcomes (22/46 (48%) vs. 3/30 (10%), *p* = 0.0004), surrogate kidney outcomes (46/46 (100%) vs. 24/30 (80%), *p* = 0.003), and surrogate cardio-respiratory (31/46 (67%) vs. 9/30 (30%), *p* = 0.001). Low-quality studies more frequently reported clinical side effects (which were mainly procedural complications) than high-quality studies (11/30 (37%) vs. 8/46 (18%), *p* = 0.04).

The reporting frequency of individual clinical, surrogate, and patient-centered outcomes is given in Table 2. Physical development was assessed nearly exclusively by height and weight, with no reports on stages of pubertal development. Only 3 studies (4%) assessed renal osteodystrophy or bone metabolism, but only by measuring parathyroid hormone or mentioning the diagnosis of hyperparathyroidism. No studies assessed bone density measures, vitamin D levels, or bone fractures.

**Table 2:**
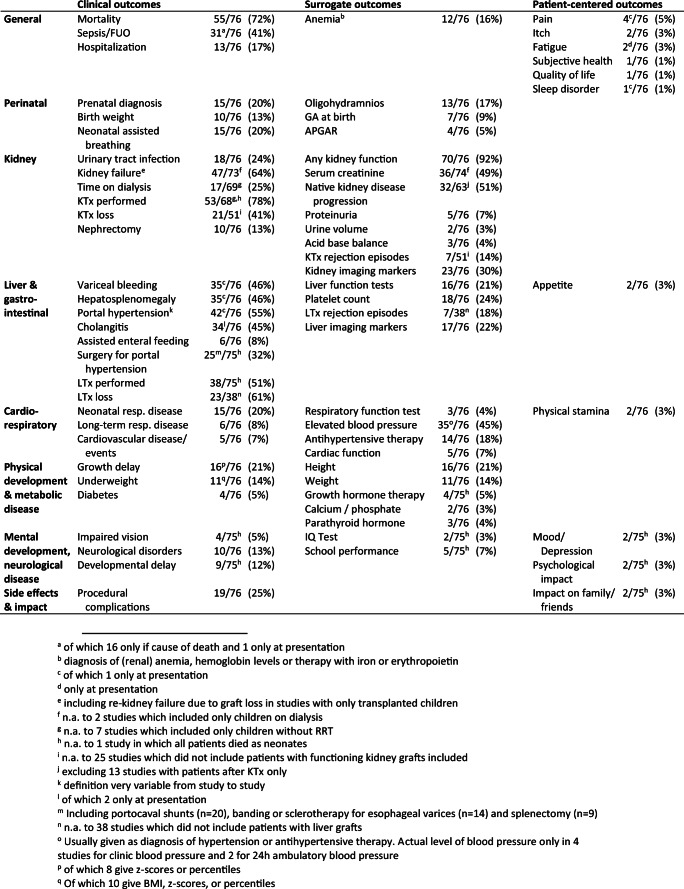
Number of ARPKD studies since 1990 reporting individual clinical, surrogate, and patient-centered outcomes. *FUO* fever of unknown origin, *GA* gestational age, *IQ* intelligence quotient, *KTx* kidney transplant, *LTx* liver transplant, *resp* respiratory

### Outcomes by importance to stakeholders

Of the outcomes which were of *core importance* to all stakeholders in the SONG-Kids project (which are life participation, survival, kidney function, and infection), kidney function was the most universally reported in 92% of studies (70/76). However, only 32/63 (51%) included an indication of the evolution of ARPKD-induced loss of kidney function over time (e.g., GFR slope or time to kidney failure). Only 9/13 (69%) studies with exclusively post–kidney transplant patients indicated the evolution of transplant kidney function over time.

Most studies reported on infections 57/76 (75%); however, this was often only one specific type of infection such as cholangitis (34/76, 45%), urinary tract infection (18/76, 24%), peritonitis in dialysis patients (3/69, 4%), or post-transplant opportunistic infections (4/69, 6%). Sepsis or fever of unknown origin was reported in 31/76 (41%) of studies, but mostly (18/31, 58%) only if it was a cause of death or presentation, rather than a time-specific incidence.

Patient survival was reported in 55 (72%) of all studies and in 48 of 58 longitudinal studies (83%).

Reports on life participation were reported the least frequently with only 4/76 studies (5%) reporting on schooling, 9/76 (12%) on the diagnosis of developmental delay and only 2/76 (3%) quantifying developmental, psychological, or behavioral problems with the help of formal scales. Overall, 16/76 (21%) of studies reported any measure of cognition, school, vision, psychological impact, or impact on family or friends.

**Fig. 2:**
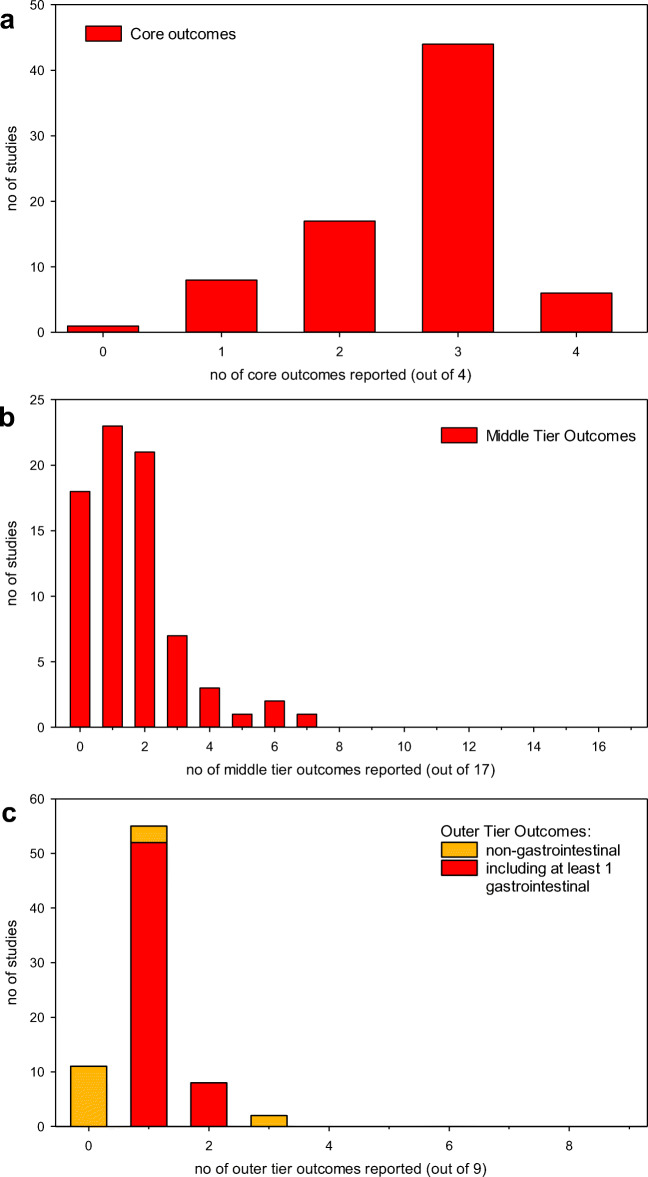
Number of **a** core, **b** middle-tier, and **c** outer-tier outcomes covered in 76 clinical studies on ARPKD since 1990

Most studies reported at least 2 of the 4 SONG-kids *core outcomes* (see Fig. 2a), with a mean of 2.6 ± 0.8 domains covered per study and only 1 study reporting no core outcome measure.

Of the 17 outcomes of the SONG-Kids *middle tier* (which are ability to travel, work, academic performance, anemia, anxiety/stress, blood pressure, bone health, cardiovascular disease, cognition, depression, fatigue, growth, hospitalization, impact on family and friends, mobility, pain, self-esteem), most studies covered between 1 and 2 areas (mean 1.63 ± 1.5) and 17 reported on none (see Fig. 2b).

Of the 9 outcomes of the *outer tier* (which are appearance, diabetes, financial impact, gastrointestinal problems, itching, sleep, thirst appetite, and vision) most studies covered only one, with a mean of 1.0 ± 0.6 outcome domains reported (see Fig. 2c). This was mainly due to the reporting of gastrointestinal problems in 62/76 (82%) of studies.

### Patient-reported outcome measures and subjective perspectives

Only 11 of 76 studies (15%) examined any patient-centered outcome and only 2 studies used validated instruments for this purpose. No individual patient-centered measure was reported in more than 5% of studies. Symptoms like pain [6, 20–22], fatigue [21, 23], sleep disorder [6], itching [20, 24], or loss of appetite [20, 25] were mentioned mostly in narrative fashion in individual studies but not evaluated or graded systematically by PROMs. There were no studies that reported the outcomes of ability to work, ability or travel, self-esteem, appearance, or financial impact.

Health-related quality of life was only examined systematically in a single study [26], using the PedsQL™ instrument. However, this study of children after combined liver and kidney transplantation did not report results for the ARPKD subgroup (10 of 23 patients) separately. Another study asked 126 children and adults with pediatric-onset portal hypertension (of which only 7% had ARPKD) to rank their general health on a numeric scale of 1 to 10, but it is unclear how many of the 65 respondents had ARPKD [27].

While 9 studies mentioned individual patients with the diagnosis of developmental delay, only one of these used systematic assessments [28]. This study in 22 children with ARPKD from the CKiD cohort [28] is noteworthy for its formalized testing of intellectual functioning, academic achievement, attention regulation, executive functioning and behavior, and comparison to children with other causes of CKD as controls.

Patient perspectives on their disease were also only reported in extremely few studies: a study on pregnancy outcomes in women affected by ARPKD included some narrative references to reasons for their reproductive choices [29]. A further study, which is not included here because it did not report any clinical outcomes, interviewed adults considering living related liver donation for a child [30]. However, less than 6 of 81 children were affected by ARPKD. There were no studies reporting treatment satisfaction or caregiver burden.

## Discussion

Overall, the body of robust data on clinical outcomes in ARPKD is still small. Scientific investigations are still endeavoring to understand underlying pathophysiology and genetic aspects of the disease, and due to the current lack of specific therapeutic options, there are few controlled trials. Even though two-thirds of the published original studies on ARPKD since 1990 assessed human outcome data, this was very often only based on very small case series. The sparsity of patient numbers together with the severity of the multi-organ disease make the attribution of cause and effect of different interventions difficult. Of the 76 studies included here, many were small to medium case series, illustrating the difficulties of systematic clinical research in very rare diseases which requires the cooperation of multiple centers of expertise. International registries are an important step forward in this scenario [31] and may help to validate different PROMs, e.g., with respect to their correlation to more traditional measures of disease severity.

The outcome domains covered in clinical studies showed a slight preponderance of reporting renal over hepatic outcomes. This probably reflects the fact that kidney disease often presents earlier than liver disease and pediatric nephrologists are therefore often primary treating physicians. Also, the increasing success of pediatric kidney replacement therapy and transplantation has been the basis for larger number of patients surviving to experience more advanced liver disease. Finally, awareness of older patients with a dominant hepatic phenotype has only grown after more widespread genetic testing became available and these patients were probably often unidentified in the past.

Surrogate outcomes were used much more frequently to assess renal outcomes than hepatic outcomes, reflecting the fact that kidney function is defined largely via laboratory tests while there are ongoing difficulties to define and quantify portal hypertension with very heterogeneous clinical and surrogate methods of assessment. These difficulties probably also explain why high-quality studies (which reported results systematically and separately for ARPKD patients, if they were a subgroup) reported significantly more often from the domains of perinatal, kidney, and cardio-respiratory outcomes, but not more often on gastrointestinal issues.

When the ARPKD studies reviewed here are compared to a survey of randomized trials in children with CKD of all causes, ARPKD studies more often covered the domains of kidney function (92% vs. 32%), infection (75% vs. 32%), and mortality (72% vs. 14%) [15]. This probably reflects the generally more severe course of children with ARPKD with earlier onset of kidney failure, higher mortality, and additional risk of cholangitis compared to the general pediatric CKD population. Measures of blood pressure (45% vs. 37%) and quality of life (1% vs. 1%) were reported at similar frequency in ARPKD studies and randomized pediatric CKD trials [15].

There was a striking lack of integrating patients into reporting of symptoms as well as assessment of neurological damage and psychological effects of the disease. Even fewer studies captured patient-centered outcomes systematically using PROMs. This is probably due to several circumstances: firstly, ARPKD is evidently a severe disease with drastic outcomes such as complete loss of kidney function, long times of hospitalization and significant mortality, which can be “easily” measured. Even though these outcomes are obviously highly relevant to the patients and their families, an exclusive focus on these endpoints may produce the illusion that explicitly quantifying patients’ subjective symptoms is unnecessary. Secondly, patient-centered outcomes are difficult to collect retrospectively because they are usually recorded unsystematically in clinical records. Prospective and systematic evaluation requires careful selection of survey tools and is often time-intensive to apply. This is illustrated by the fact that procedural complications were mentioned more frequently in low-quality studies, as it is easier to recount them in narrative case reviews than to capture them systematically. Finally, patient-centered outcomes are more difficult to elicit from young children than adults, and infancy is a time of many severe manifestations of ARPKD. Even though capture via parent-proxy instruments is possible, there are generally less validated tools for children than adults.

While patient-reported outcomes are of course not an end in themselves, the key outcomes for children with CKD which were prioritized after extensive multi-stakeholder discussions of the SONG-Kids project (e.g., life participation, anxiety/stress or pain) necessarily require to elicit the patient’s (or a proxy’s) perspective. When comparing the four outcome areas that were of key importance to all stakeholders, kidney function, infection, and survival were covered by most studies, but life-participation only in a small minority. It is therefore fair to say that the patients’ and parental perspectives are our “blind spot” in clinical studies on ARPKD and should be represented better in future studies.

Measuring studies up to the core outcomes defined by the SONG-Kids project appeared most appropriate to us, as there is no qualitative research on patient perspectives in ARPKD so far and the parallel SONG-PKD project focuses on adults with ADPKD. This is reflected by many adult concerns (e.g., fertility, sexual function, cardiovascular disease) and issues specific to ADPKD (e.g., cyst bleeds, cyst infections, and cyst growth) in the SONG-PKD core outcome set [19]. However, SONG-Kids is also not entirely appropriate to children with ARPKD, as they more frequently have hepatic involvement and often suffer from stage 5 chronic kidney disease earlier than children with other causes of CKD. Gastrointestinal problems are therefore likely to feature higher on their list of priorities than in SONG-kids, where these are only part of the outer-tier outcomes. We therefore think additional studies to capture patient and family perspectives of ARPKD would be very valuable to assess the impact of multi-organ involvement as well as the younger age and more severe course of kidney disease compared to the general pediatric CKD cohort. In an ongoing study, we aim to elicit patients’ and carers’ priorities regarding disease outcomes in ARPKD and validate existing PROMs by correlation to indicators of disease severity [32].

Our attempts to quantify for each study how many of the SONG-Kids outcomes were covered were limited by the fact that “life participation” is a vague term that includes many of the outcomes that are part of other tiers such as school attendance, academic achievement, or impact on family and friends. It is unclear whether developmental delay is part of the concept of life participation, as it may well impair academic achievement and job prospects but not necessarily engagement in social interaction. There is no established PROM for capturing life participation as a whole, and the relatively few pediatric CKD studies that attempt to capture it use a wide variety of different instruments [33]. Indeed, the plethora of PROMs (mostly questionnaires) for measuring closely related concepts such as health-related quality of life can be quite overwhelming, while simultaneously only few have good data for children with kidney diseases. Further studies to standardize and validate measures of life participation for children with CKD are therefore needed. Development of electronic versions and mobile health apps for these instruments may help to simplify their integration into clinical trials by reducing administrative effort and encouraging patient participation.

We propose that future studies in ARPKD place higher emphasis on patient-centered outcomes, either by using existing validated measures of health-related quality of life in children (with CKD) in addition to traditional clinical and surrogate outcomes or by focusing on developing and validating new disease-specific PROMs that reflect the multi-organ involvement of ARPKD. Even though this usually increases the administrative effort in clinical trials, it will help to make them more relevant not only to patients but also to healthcare providers.

A limitation of this study is that outcome parameters within an area were not further ranked by relevance. However, as in pediatric studies on CKD in general [15], there was large heterogeneity with regard to exact outcome parameters used for one area, and therefore ranking would have been very subjective. Also, our aim was not to criticize the way that outcome data are recorded, but to highlight the lack of patient-centered outcomes in ARPKD studies to date.

We do not wish to suggest that quantifying how many outcome areas are covered in a study gives full information about whether the study was well-conducted or produced meaningful results. For example, a study with a clear focus and appropriate control group may be able to answer many more questions than a narrative description of a much greater number of patients. Also, failure to report an outcome area, e.g., stages of puberty, may be excusable for most studies on ARPKD but is a flaw in a study examining treatment with growth hormone in ARPKD. Other outcomes, such as surgical treatment of portal hypertension, are relevant for most patients with ARPKD, but will not be applicable in a study focusing on neonatal outcomes.

In conclusion, even though ARPKD is a rare disease with many outcomes that are evidently undesirable for patients, further efforts are needed to elicit patient and family perspectives on the disease and to develop systematic tools to integrate patient-centered outcomes into clinical studies.

## Supplementary Information

Graphical abstract (PPTX 277 kb).

## Data Availability

The datasets generated during the current study are available from the corresponding author on reasonable request.

## Footnotes

1 Defined as systematic and ARPKD-specific reporting; see “Methods” section.
