# Supplementary material for: Systematic review on outcomes used in clinical research on autosomal recessive polycystic kidney disease—are patient-centered outcomes our blind spot?
Source: Pediatr Nephrol. 2021 Aug 12;36(12):3841–51. doi: 10.1007/s00467-021-05192-8 (PMC8599334; doi:10.1007/s00467-021-05192-8)
Supplement: Supplementary file 1 — (PPTX 277 kb). [file 467_2021_5192_MOESM1_ESM.pptx]

## Slide 1
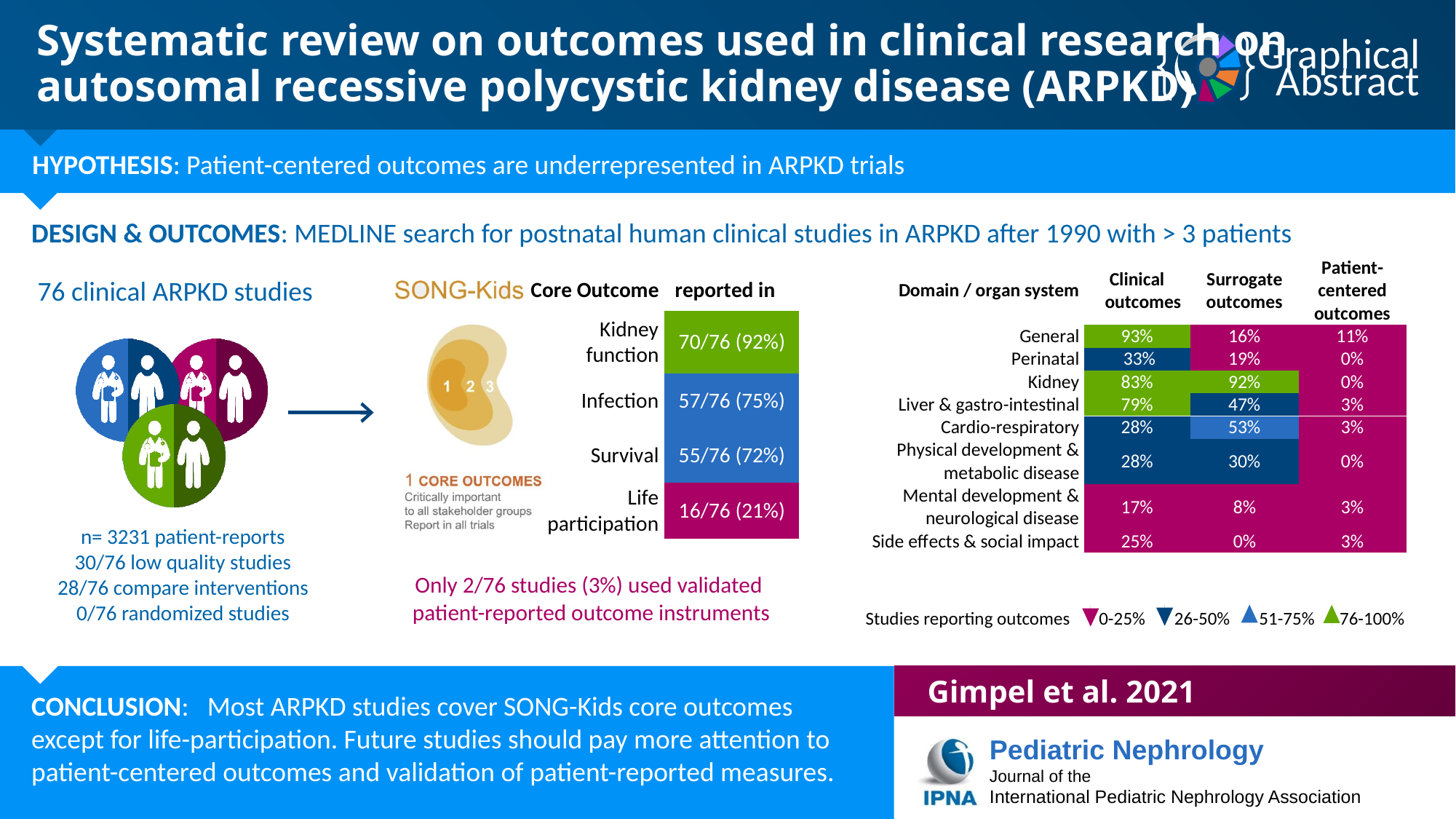

Systematic review on outcomes used in clinical research on
autosomal recessive polycystic kidney disease (ARPKD)
HYPOTHESIS: Patient-centered outcomes are underrepresented in ARPKD trials
DESIGN & OUTCOMES: MEDLINE search for postnatal human clinical studies in ARPKD after 1990 with > 3 patients
 76 clinical ARPKD studies
n= 3231 patient-reports
30/76 low quality studies
28/76 compare interventions
0/76 randomized studies
Only 2/76 studies (3%) used validated
patient-reported outcome instruments
Studies reporting outcomes 0-25% 26-50% 51-75% 76-100%
Gimpel et al. 2021
CONCLUSION: Most ARPKD studies cover SONG-Kids core outcomes except for life-participation. Future studies should pay more attention to patient-centered outcomes and validation of patient-reported measures.
